# Reactivity of Curcumin: Theoretical Insight from a Systematic Density Functional Theory-Based Review

**DOI:** 10.3390/ijms262110374

**Published:** 2025-10-24

**Authors:** Marcin Molski

**Affiliations:** Department of Quantum Chemistry, Faculty of Chemistry, Adam Mickiewicz University of Poznań, ul. Uniwersytetu Poznańskiego 8, 61-614 Poznań, Poland; mamolski@amu.edu.pl; Tel.: +48-602-250-063

**Keywords:** curcumin reactivity, DFT calculations, thermodynamic descriptors, dispersion effect, radical scavenging, keto–enol tautomerism, di-curcumin derivative

## Abstract

A comprehensive analysis of key findings derived from density functional theory (DFT) studies reveals that current theoretical data on curcumin remain incomplete, underscoring the need for further computational investigation to achieve a more thorough understanding of its chemical and biological reactivity. This study addresses these gaps through four primary objectives: (i) determination of a complete set of thermodynamic descriptors and elucidation of the multi-step anti-radical mechanisms of the neutral, radical, anionic, and radical–anionic forms of curcumin; (ii) calculation of global chemical reactivity descriptors of curcumin in various solvent environments; (iii) theoretical reproduction of experimentally determined p*K*_a_ values for all active sites within the molecule; and (iv) examination of the effects of dispersion interactions and solvent polarity on the reactivity descriptors of keto–enol forms of curcumin. The results obtained provide enhanced insight into the molecular behavior of curcumin, facilitating improved predictions of its reactivity under diverse conditions. Moreover, the findings indicate a potential structural modification of the keto form of curcumin, involving the attachment of two 4-hydroxy-3-methoxyphenyl-prop-1-en-2-one moieties to the methylene group. The resulting modeled compound, referred to as di-curcumin, exhibits enhanced chemical reactivity and increased anti-radical potential.

## 1. Introduction

Curcumin, the principal curcuminoid derived from the rhizome of *Curcuma longa*, has been the focus of intense scientific inquiry owing to its pleiotropic biological activities and its potential as a versatile therapeutic agent [1,2,3,4,5]. Historically utilized in traditional medicine systems such as Ayurveda and traditional Chinese medicine, curcumin has, in recent decades, garnered substantial interest within the biomedical and chemical sciences. Structurally characterized as a diarylheptanoid bearing both enol and keto functional groups, curcumin exhibits remarkable chemical reactivity that underlies its diverse bioactivities, including anticancer, anti-inflammatory, antimicrobial, antiradical, photoprotective, and wound healing effects [6,7,8,9,10,11,12,13,14,15,16,17,18].

Contemporary experimental research has increasingly turned to computational methods, particularly density functional theory (DFT), time-dependent DFT (TD-DFT), molecular docking and dynamics simulations, to elucidate the structural underpinnings of curcumin’s bioactivity [19,20,21,22,23,24,25,26,27,28,29,30,31,32,33,34,35,36,37]. These studies have yielded rich insights into its radical scavenging behavior, electronic transitions, spectral and HOMO-LUMO characteristics, as well as binding affinities toward biomolecular targets such as enzymes, DNA, and cellular receptors. Moreover, the synthesis of curcumin analogs [17,29,30,33,36] and metal-curcumin complexes [26] has emerged as a fruitful strategy to improve its solubility, stability, and bioavailability—limitations that have historically constrained curcumin’s clinical translation [13,14,15,16,18].

Numerous studies have employed the DFT method to elucidate the antiradical properties of curcumin associated with the tautomeric structure–activity relationship. Jovanovic et al. [19] demonstrated that the keto–enol equilibrium within the heptadienone moiety plays a pivotal role in determining the physicochemical and antiradical characteristics of curcumin. Under neutral and slightly acidic aqueous conditions (pH 3–7), the keto form predominates, enabling curcumin to function as a hydrogen atom donor. The calculated reaction rate constant with the methyl radical, (3.5 ± 0.3) × 10^9^ M^−1^ s^−1^, approaches the diffusion-controlled limit in 40% aqueous DMSO at pH = 5 [19]. In contrast, the tert-butoxyl radical reacts with curcumin in acetonitrile at a rate constant of (7.5 ± 0.8) × 10^9^ M^−1^ s^−1^ [19]. The principal site of reactivity was identified as the central methylene (CH_2_) group within the heptadienone linker, which bears two labile hydrogen atoms. A subsequent DFT investigation by Sun et al. [20] corroborated that the primary mechanism of radical scavenging in curcumin involves hydrogen abstraction from the phenolic OH groups, rather than from the central methylene CH_2_ group. Calculated bond dissociation enthalpies (BDE) indicated that the two phenolic moieties act independently in radical scavenging processes. Building on these findings, Shen and Ji [21] employed continuum solvent models to show that although the deprotonated forms of curcumin exhibit enhanced reactivity toward radicals, they are minimally populated under neutral pH conditions. These results underscore the relevance of the neutral form of curcumin in physiological environments and highlight that the stabilization of phenoxyl radicals is strongly influenced by intramolecular hydrogen bonding and extended π-conjugation, which facilitate effective delocalization of unpaired electrons. Manzanilla and Robles [22] conducted a comparative study involving curcumin, caffeic acid phenethyl ester, and chicoric acid, identifying hydrogen atom transfer (HAT) as the most favorable antiradical mechanism. Similarly, Purushothaman et al. [23] examined structural analogs such as polyhydroxycurcumin and hispolon, demonstrating that dihydroxy substitution enhances antioxidant activity via reduced BDE values, particularly under oxidative stress conditions involving hydroxyl and peroxyl radicals. More recently, Biswas and Shukla [24] investigated the scavenging activity of curcumin R–H toward the methyl and ethyl radicals X^●^ through radical adduct formation (RAF), HAT and single electron transfer (SET) mechanisms specified below:

R–H + X^●^ → R–XH^●^ (RAF)

R–H + X^●^ → R^●^ + X–H (HAT)

R–H + X^●^ → R–H^●+^ + X^−^ (SET)

The calculations demonstrated that the curcumin can scavenge methyl radicals through both RAF and HAT, preferring RAF over the HAT, whereas the SET scenario has been found to be highly endergonic and so not viable. A short review of computational studies performed so far reveals a predominant focus on the anti-radical activity of the neutral keto–enol forms of curcumin. However, Galano et al. [25] emphasized the necessity of extending this analysis to include the anionic species formed through the three-step deprotonation of curcumin in hydrophilic environments. This observation underscores the potential involvement of all active sites of the molecule in its overall reactivity. Furthermore, given that radical species of curcumin can be generated in hydrophobic media, a comprehensive evaluation of curcumin’s reactivity must encompass the anionic, radical, and radical–anionic forms of this compound. In addition to determining thermodynamic characteristics and elucidating the mechanisms underlying curcumin’s activity across different solvent environments, it is also essential to compute global chemical reactivity descriptors. The relevance of these parameters was demonstrated by Murakami et al. [26] in a comparative study of curcumin and tetrahydrocurcumin activity. Their findings highlight the value of such descriptors as a complementary tool in the thermodynamic characterization of the compounds, enhancing both the interpretation and prediction of their chemical behavior.

One of the key descriptors of molecular reactivity is the acid dissociation constant (p*K*_a_), which reflects a compound’s propensity to undergo deprotonation in solutions of varying polarity and pH. The p*K*_a_I, p*K*_a_II, and p*K*_a_III values for curcumin have been determined experimentally, with slight variations depending on the methodologies and experimental conditions employed. For instance, reported values in aqueous solution include 7.75–7.80, 8.55, and 9.05 [38], as well as 8.38, 9.88, and 10.51 [39]. In a 1:1 water/methanol mixture, values of 8.54 ± 0.03, 9.30 ± 0.03, and 10.69 ± 0.02 have also been reported [40]. Additional measurements include values of 8.55 and 10.41 [19] and 8.10 and 10.15 [41]. These experimental findings have prompted ongoing discussion regarding the sequential deprotonation of hydroxyl groups in the enolic form of curcumin. According to Shen and Ji [21], the first deprotonation involves the enolic hydroxyl proton, followed by the phenolic hydroxyl groups. The p*K*_a_ value for the enol group calculated using DFT and TD-DFT methods was 8.8 and 9.4 [21,42]. In contrast, Lestari and Indrayanto [42] suggest that the first two p*K*_a_ values correspond to the deprotonation of the phenolic hydroxyl groups, while the third pertains to the enolic proton. The matter remains open and still awaits explanation.

The theoretical reproduction of experimental p*K*_a_ values with accuracy comparable to standard experimental error remains a significant challenge for computational methodologies, particularly the DFT approach [43,44,45]. To this aim, the various solvation models, with particular emphasis on the Conductor-like Polarizable Continuum Model (C-PCM) [46], the Integral Equation Formalism Polarizable Continuum Model (IEF-PCM) [47], and the universal Solvation Model Density (SMD) based on solute electron density [48] are employed. For monoprotic compounds, DFT-based calculations including local spin density approximation (LSDA) [49] and Becke 3-parameter Lee–Yang-Parr (B3LYP) functionals [50,51] generally achieve a predictive accuracy within the range of 0.2–0.5 p*K*_a_ units [43,44,45]. However, in the case of multiprotic systems, accurate prediction necessitates the application of extended basis sets and more sophisticated computational approaches [52] to account for the complex interplay of protonation states and solvation effects [53,54,55,56]. In this respect, Manolova et al. [55] used UV-Vis spectroscopy and quantum-chemical calculations to show that in pure ethanol, curcumin exists exclusively in its enol form, whereas increasing water content favors the keto form. Theoretical modeling demonstrated [55] that water molecules stabilize the keto tautomer via hydrogen bonding, providing a rare quantitative view of tautomeric shifts in polar environments. Given that the equilibrium between the keto–enol tautomeric forms of curcumin is influenced by solvent polarity [56], it is essential to consider both tautomers when determining the reactivity descriptors of curcumin in solvents spanning a broad range of dielectric constants. This thesis is supported by the study conducted by Madinah et al. [57], which demonstrated that a comprehensive understanding of the complex influence of solvent polarity on keto–enol tautomeric equilibrium requires consideration of noncovalent interactions, particularly dispersion forces. These interactions significantly contribute to the stability of flexible molecules, including curcumin. Furthermore, their study proposes a water-catalyzed mechanism for tautomerization, wherein the inclusion of dispersion corrections reduces the energy barrier for keto–enol interconversion and renders the keto form both thermodynamically and kinetically favorable. The analysis conducted using the B3LYP functional in conjunction with the D3 empirical dispersion correction, as well as the Austin–Frisch–Petersson (AFPD) functional incorporating spherical atom dispersion terms [58], revealed that the inclusion of dispersion interactions significantly influenced the ground-state optimized structure of curcumin. This led to enhanced stability and notable modifications in molecular geometry. Given the critical role of dispersion in mediating intramolecular noncovalent interactions, these results strongly indicate that dispersion effects must be considered in studies of curcumin reactivity, even under gas-phase conditions [57]. It is noteworthy that the study by Madinah et al. [57] did not examine the influence of dispersion interactions on the anti-radical activity of curcumin, thereby identifying a significant gap in the existing literature and an avenue for future research. Furthermore, more advanced functionals—such as this developed by Head-Gordon and collaborators [59], which incorporates Grimme’s D2 dispersion model—were not employed in the investigation [57].

The brief overview presented above summarizes the most significant findings from theoretical studies conducted to date on the reactivity of curcumin using DFT methodology. This synthesis highlights several areas where further investigation is warranted. Such research is essential for a comprehensive understanding of the structure–medium–activity relationship of curcumin, which underpins its potential applications in the medical, cosmetic, and food industries. Accordingly, the present study is designed to address four principal objectives: (i) to determine a comprehensive set of thermodynamic descriptors and elucidate the corresponding anti-radical activity mechanisms of the neutral, radical, anionic, and radical–anionic keto–enol forms of curcumin; (ii) to compute global chemical reactivity descriptors of curcumin in various solvents in order to identify those that enhance its reactivity; (iii) to theoretically reproduce the experimentally determined p*K*_a_ values for all active sites of curcumin with an accuracy comparable to the experimental uncertainties; and (iv) to examine the influence of dispersion effects coupled with solvent polarity on the reactivity of curcumin in its keto–enol tautomeric forms.

## 2. Results and Discussion

To achieve the primary objectives of this work, the thermodynamic and global chemical activity descriptors of curcumin in the keto–enol forms have been determined. For this purpose, the following quantities defined in the Supplementary Materials are taken into account:

(i) bond dissociation enthalpy BDE, adiabatic ionization potential AIP, proton dissociation enthalpy PDE, proton affinity PA, and electron transfer enthalpy ETE [60,61,62,63] in the gas phase, hydrophilic (water) and hydrophobic (benzene) solvents;
(ii) the ionization potential IP, electron affinity EA, energy gap ∆E, chemical potential μ, absolute electronegativity χ, molecular hardness η and softness S, electrophilicity index ω, the electro-donating ω^−^ and electro-accepting ω^+^ powers, and the Ra, Rd indexes [63,64,65,66,67,68,69,70].

**Figure 1:**
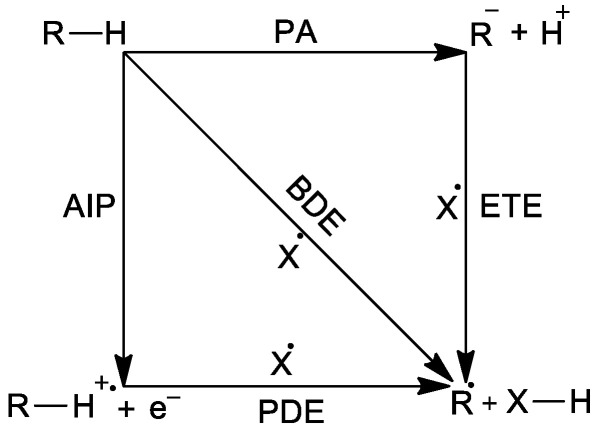
The thermodynamic descriptors associated with the radical deactivation mechanisms: BDE for hydrogen atom transfer (HAT), AIP and PDE for single electron transfer followed by proton transfer (SET-PT), PA and ETE for sequential proton loss electron transfer (SPLET).

The radical scavenging efficacy of the compounds investigated is determined by thermodynamic descriptors associated with the following deactivation mechanisms: BDE for hydrogen atom transfer (HAT), AIP and PDE for single electron transfer followed by proton transfer (SET-PT), PA and ETE for sequential proton loss electron transfer (SPLET). These descriptors, as related through the scheme presented in Figure 1, can be computed using the enthalpies of the cation H(R–H^●+^), radical H(R^●^), anion H(R^−^), the parent compound H(R–H), and the enthalpies of hydrogen H(H^●^), the electron H(e^−^), and the proton H(H^+^).

**Figure 2:**
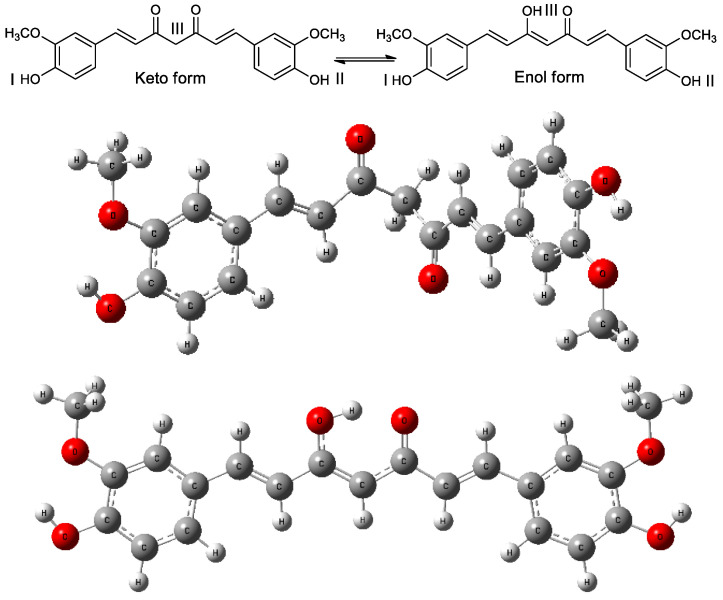
The optimized geometries of the lowest-energy keto–enol tautomers of curcumin and potential sites I, II and III of deprotonation or dehydrogenation. The calculations were performed in the gas phase at the B3LYP/6311++G(d,p) theory level.

A lower value of the calculated parameter indicates lower energy requirements for dehydrogenation (HAT), ionization (SET-PT), and deprotonation (SPLET) in the initial phase of radical deactivation. For two-stage processes, the sum of the relevant parameters (PA + ETE or AIP + PDE) should also be considered. The geometries of all compounds, including their cationic, anionic, and radical forms in the various media with different dielectric constants, were optimized following the procedure described in the Materials and Methods Section. To fulfill the primary objectives of this study, the initial stage involved the analysis of various conformers and rotamers of the keto–enol tautomers of curcumin, with the aim of selecting the most stable structures for the generation of anionic, cationic, and radical species of the parent compound. The total electronic energies and optimized geometries of the neutral structures in the gas phase are presented in Figure S1. The lowest-energy keto (E_1_ = −1263.932619 Ha) and enol (E_2_ = −1263.943446 Ha) tautomers, identified through this analysis, were subsequently employed for the calculation of thermodynamic and chemical reactivity descriptors in both the gas phase and solvents of varying polarity. The majority of both theoretical and experimental investigations recognize these two conformers as representative forms of the keto and enol tautomers [71,72,73,74,75,76,77]. The computational protocol considered three potential sites of deprotonation (dehydrogenation): the three hydroxyl groups I, II, III in the enol tautomer, as well as two hydroxyl groups I, II, and the central methylene hydrogen III in the keto form presented in Figure 2.

### 2.1. Dispersion Effect

Building upon the findings of Madinah et al. [57], the present study examined the influence of dispersion interactions on the relative energies of curcumin tautomers in both the gas phase as well as aqueous and benzene solutions. The results are reported in Table 1.

The computational analysis demonstrated that the inclusion of dispersion corrections results in the stabilization of the conformers by lowering their total energies and diminishing the energy difference between the keto and enol forms in all phases considered. Notably, the energy difference of 0.45 kcal mol^−1^ falls below the generally accepted threshold 1 kcal mol^−1^ for thermochemical accuracy of calculations. These findings indicate that, within the framework of the dispersion-corrected DFT/ωB97XD-D2 model, the keto and enol tautomers of curcumin in aqueous solution exhibit comparable energies. This suggests that, contrary to the prevailing assumption, the enol form is not energetically favored in water. In contrast, a discernible energy difference between the two tautomers is observed in the gas phase and benzene medium.

### 2.2. Thermodynamic Descriptors

Optimized keto–enol structures of curcumin were employed to calculate the enthalpies of radicals, cations, and anions derived from the parent compound at individual active sites I, II, and III. These calculations were conducted in the gas phase, as well as in benzene (ε = 2.247) and water (ε = 78.39), the latter two solvents serving as models for hydrophobic and hydrophilic environments, respectively. Dispersion interactions were also incorporated to assess their influence on the thermodynamic parameters of curcumin, particularly in aqueous solution. The computational results, summarized in Table 2, indicate that for the keto tautomer, the hydroxyl groups at positions I and II exhibit equal (or approximately equal) enthalpy values, i.e., they are equienergetic.

In contrast, in the enol form, these two sites display a slight difference in enthalpy, approximately 0.5 kcal mol^−1^. In particular, in water, PA_I_ + ETE_I_ = 121.75, PA_II_ + ETE_II_ = 122.33 kcal mol^−1^, which equalize after inclusion dispersion, reaching values of PA_I_ + ETE_I_ = 122.26 and PA_II_ + ETE_II_ = 122.19 kcal mol^−1^. Notable exceptions include the BDE for sites I and II in benzene, which differ by 1.40 kcal mol^−1^, and the PA for the same sites in the gas phase, where the difference is 1.1 kcal mol^−1^. The results presented in Table 2 also reveal that the inclusion of dispersion in the calculations alters the thermodynamic descriptors of all active sites in the enol form, as well as sites I and II in the keto form, by an average of approximately 0.5 kcal mol^−1^. An exception is observed only for the mobile hydrogen of the central methylene group, for which the PA_III_ increases from 43.05 to 44.36 kcal mol^−1^ (ΔPA_III_ = 1.31 kcal mol^−1^), indicating a stabilizing effect of dispersion on the keto form of the compound under investigation. Importantly, since the relative order of proton affinities remains unchanged (PA_I_ ≈ PA_II_ < PA_III_), the inclusion of dispersion does not influence the identification of the preferred free radical scavenging scenario. In the gas phase and in benzene, the mechanism proceeds via HAT, whereas in water, it follows the SPLET pathway. Furthermore, the relatively small variation (≈0.5 kcal mol^−1^) observed in the remaining parameters suggests that the dispersion effect can be reasonably neglected when computing thermodynamic descriptors BDE, PA, ETE, AIP, PDE for I and II centers of the keto-form and all sites of the enol tautomer. The underlying reason is that this effect is partially canceled during the calculation of descriptors that are defined based on enthalpy differences among the neutral, radical, cationic, and anionic species, as outlined in the Supplementary Materials. The magnitude of the dispersion effect can be quantitatively estimated using the enthalpy differences ∆H(x) provided in Table 3, which facilitates the determination of changes in the descriptor values for the keto form of curcumin in aqueous solution under the influence of dispersion.

For example (values in kcal mol^−1^): ∆BDE_I_ = ∆H(N) − ∆H(R_I_) = 0.5685, ∆PA_I_ = ∆H(N) − ∆H(A_I_) = 0.5635, ∆ETE_I_ = ∆H(A_I_) − ∆H(R_I_) = 0.0050, ∆AIP = ∆H(N) − ∆H(C) = 0.1995, and ∆PDE_I_ = ∆H(C) − ∆H(R_I_) = 0.7680.

Calculations performed for the keto form of curcumin with a single water molecule placed at active sites I, II, and III yielded the following values of the descriptor (with values in parentheses corresponding to the system without the water molecule): BDE_I_ = 77.36 (77.38), BDE_II_ = 77.45 (77.38), BDE_III_ = 86.92 (87.11), PA_I_ = 39.49 (40.87), PA_II_ = 39.48 (40.88), PA_III_ = 41.59 (43.05), ETE_I_ = 83.97 (82.60), ETE_II_ = 84.07 (82.59), ETE_III_ = 91.42 (90.15), PDE_I_ = 19.06 (19.00), PDE_II_ = 19.15 (19.00), PDE_III_ = 28.51 (28.73), AIP = 104.40 (104.47) kcal mol^−1^. The results indicate that calculations performed at the B3LYP/6-311++G(d,p) level of theory, employing the SMD solvation model in an aqueous environment, yield comparable descriptor values and do not significantly influence the interpretation of the obtained results. The most pronounced differences are observed in the PA and ETE parameters, where the reduction in their values suggests a weakening of the OH and CH group bonding, as well as the activation of the SPLET mechanism induced by the presence of the water molecule.

### 2.3. Radical Scavenging Mechanisms

The thermodynamic descriptors presented in Table 2 facilitate the identification of the most favorable radical deactivation mechanism. In the gas phase and in benzene, the HAT pathway is preferred, whereas in an aqueous environment, the SPLET scenario is favored. This mechanistic assignment is consistent across all active centers and is based on the activity paradigm proposed by Sun et al. [20]. According to this model, the antiradical activity of curcumin is attributed to the hydroxyl groups at centers I and II, which operate independently via either the HAT or SPLET mechanism. However, this approach does not fully capture the actual chemical behavior, as dehydrogenation (deprotonation) at center I results in a radical (anionic) species that exhibits thermodynamic properties distinct from those of the neutral parent molecule. Therefore, to accurately assess the activity of center II—and subsequently center III—it is necessary to determine the thermodynamic descriptors of the species formed at each stage. To provide a consistent and unambiguous framework for describing the possible reaction pathways, the following notation is introduced: N denotes the neutral, C the cationic, A the anionic, and R the radical forms, respectively. The activity centers I, II, and III correspond to the sequential positions in the notation, indicating the specific site of reaction. For example, curcumin in its fully neutral state is represented as NNN; a radical formed via dehydrogenation at center I is denoted RNN; the corresponding anionic species generated via deprotonation is ANN; the cationic form is represented as CNN; and so forth. Assuming that curcumin initiates its activity at center I, the thermodynamic parameters presented in Table 2 enable the identification of the preferred radical deactivation mechanism. Specifically, HAT is favored in the gas and hydrophobic phase (benzene), while SPLET predominates in the hydrophilic medium (water), leading to the formation of the RNN and ANN species, respectively. These species provide the foundation for the second and third stages of reactivity, involving centers II and subsequently III. In this manner, the antiradical activity of curcumin can be described as a multistep process in which all reactive centers of both tautomers participate. The results of calculations are reported in Table 4 and Table 5.

**Figure 3:**
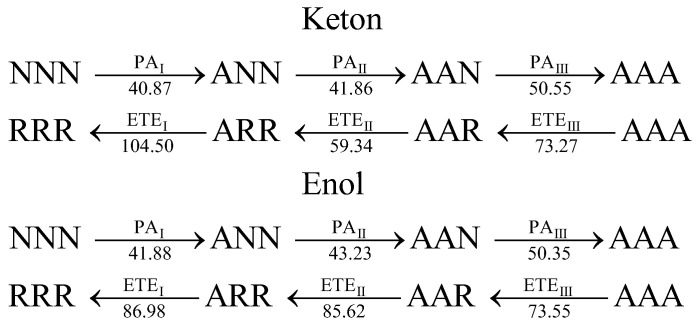
The multi-step SPLET mechanisms of radical deactivation by curcumin tautomers, including sequential deprotonation from sites I, II, and III, characterized by PA_I_, PA_II_, and PA_III_, and next, sequential radical deactivation in the reverse order, characterized by the thermodynamic descriptors ETE_III_, ETE_II_, and ETE_I_. The values of parameters (in kcal mol^−1^) are taken from Table 4 and Table 5.

**Figure 4:**
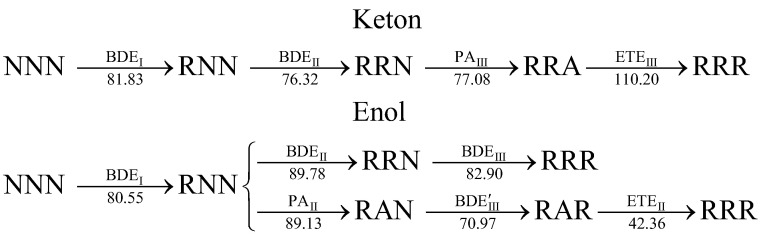
The multi-step HAT and mixed HAT-SPLET mechanisms of radical deactivation by curcumin tautomers, including sequential dehydrogenation from I, II, and III sites, characterized by BDE_I_, BDE_II_, and BDE_III_. The mixed mechanism is characterized by descriptors PA_III_, ETE_III_, PA_II_, and ETE_II_. The values of parameters (in kcal mol^−1^) are taken from Table 4 and Table 5, whereas BDE’_III_ = 70.97 and ETE_II_ = 42.36 were additionally calculated.

By applying the minimum-energy criterion, the most energetically favorable mechanisms of radical deactivation by curcumin can be identified through the selection of low-value thermodynamic descriptors. In particular, the PA values in water support a multi-step SPLET mechanism for both tautomers. This process involves sequential deprotonation at sites I, II, and III, characterized by the descriptors PA_I_, PA_II_, and PA_III_, respectively, followed by sequential radical neutralization occurring in the reverse order. The latter steps are described by the thermodynamic parameters ETE_I_, ETE_II_, and ETE_III_. Together, these parameters outline a cascade of reactions in which three radicals are sequentially neutralized via the scheme in Figure 3:

For the keto–tautomer of curcumin in benzene, the most energetically favorable radical deactivation pathway corresponds to a mixed mechanism, involving dehydrogenation at positions I and II via the HAT, followed by deprotonation at position III via the SPLET pathway. In the case of the enol tautomer, the first step involves dehydrogenation at site I. The comparable values of BDE_II_ = 89.78 kcal mol^−1^ and PA_II_ = 89.13 kcal mol^−1^ for the transitions RNN → RRN and RNN → RAN, respectively, suggest that both HAT and the initial step of SPLET are energetically viable at position II. The subsequent step proceeds via the RAN → RAR transformation, as indicated by the lowest calculated BDE_III_ = 70.97 kcal mol^−1^ in the set of descriptors. Only after this dehydrogenation step, the second stage of SPLET RAR → RRR occurs—it is characterized by the ETE_III_ = 42.36 kcal mol^−1^. A schematic representation of all proposed reaction pathways is provided in Figure 4:

### 2.4. Determination of pK_a_ Descriptor

The calculated proton affinity (PA) values for curcumin deprotonation in an aqueous environment—both for individual centers I, II, and III (i.e., NNN → ANN, NNN → NAN, and NNN → NNA) and for the sequential formation of mono- and di-anionic intermediates (NNN → ANN → AAN → AAA)—clearly indicate the preferred order of deprotonation. For both tautomers of curcumin, the deprotonation sequence proceeds from center I, followed by center II, and finally center III. This trend is supported by the relative PA values for center III, which consistently follow the order PAIII > PAII > PAI in both the ketone and enol forms (see Table 4 and Table 5). Based on the established sequence of the multi-step deprotonation process, the Gibbs free energy values for the neutral form (NNN) and the anionic intermediates (ANN, AAN, and AAA) can be computed. These values then allow for the theoretical determination of the acidity constants p*K*_a_I, p*K*_a_II, and p*K*_a_III, using the following equation [46]:

(1) $$\begin{array}{l} pK_{a}N\;=\;a\cdot pK_{a}{\left(\mathrm{the}\right)}^{c}\;=\;a{ [\frac{{\Delta G}_{N}{+\;G(H}^{+}{)}_{\mathrm{sol}}-1.8942}{1.3642}]}^{c}\\ \;{\Delta G}_{N}{{=\;G}^{-N}}_{\mathrm{sol}}-{{\;G}^{-(N-I)}}_{\mathrm{sol}}-s\;\;\;\;N\;=\;I,\;\mathrm{II},\;\mathrm{III} \end{array}$$

in which R is the gas constant, T = 298.15 [K] is the temperature, RT∙ln(10) = 1.3642 kcal mol^−1^, s = RT∙ln(2)∙ln(10) = 0.4107 kcal mol^−1^ represents a correction [78] to the energy accounting for the presence of two equivalent deprotonation centers (I and II) as well as the two hydrogen atoms in the CH_2_ group of the ketone tautomer of curcumin. In the case of the enol form, the values of the PA_I_ + ETE_I_ and PA_II_ + ETE_II_ exhibit slight differences between sites I and II; therefore, the s correction was deemed negligible and was not applied.

The value of RT∙ln(V) = 1.8942 kcal mol^−1^ stands for the correction

(2) $$G^{1\;\mathrm{mol}}{=G}^{1\;\mathrm{atm}}-1{.8942\;\mathrm{kcal}\;\mathrm{mol}}^{-1}$$

for the reference state (V = 24.46 L, T = 298.15 K, from 1 atm to 1 mol, whereas ∆G in kcal mol^−1^ denotes the Gibbs free energy of the deprotonation according to the reaction

$$\mathrm{RH}\overset{\mathrm{solvent}}{⇄}R^{-}{+H}^{+}$$

The Gibbs free energy of the solvated proton, G(H^+^)_sol_, is related to the solvation energy of the proton ∆, G(H^+^)_sol_, by the relation:

(3) $${G(H}^{+}{)}_{\mathrm{sol}}=\;\Delta G(H^{+}{)}_{\mathrm{sol}}{+\;G(H}^{+}{)}_{\mathrm{gas}}$$

in which G(H^+^)_gas_ = −6.2883 kcal mol^−1^ [79] is the free energy of a proton in the gas phase under a pressure of 1 atm. The values of ∆G_N_ calculated at the LSDA/QZVP and ωB97XD-D2/QZVP theory levels, using the SMD solvation model and the 1:1 water/methanol medium, are reported in Table 6.

In Equation (1), additional semi-empirical parameters a and c are employed to enhance the accuracy of p*K*_a_ reproduction by adjusting them to the set of experimental p*K*_a_ = p*K*_a_(exp) data and theoretical p*K*_a_(the) values. Equation (1) was validated using gallic acid [46] and allowed for the determination of the p*K*_a_ values associated with its four-step deprotonation, achieving an accuracy of 0.01 p*K*_a_ units compared to experimental accuracy of the data. Equation (1) contains three parameters*,* a, c, and G(H^+^)_sol_ the value of which is unknown. Consequently, fitting all three parameters to the available three experimental p*K*_a_ values is impossible and necessitates the use of a scanning method (a-parameter is constrained), as described in the Materials and Methods Section. The results of calculation are presented in Table 7.

The results obtained reveal that the approach proposed enables both the precise determination of the Gibbs free energy ∆G(H^+^)_sol_ of the proton solvated in the 1:1 water/methanol environment and the accurate reproduction of the experimental p*K*_a_ values by taking advantage of the LSDA/QZVP theory level, SMD solvation model, and the set of p*K*_a_ experimental data [40]. The results fall within the range of experimental errors for both tautomers, with MAE = 0.0008, NMAE = 0.0293 for the keto-form and MAE = 0.0005, NMAE = 0.0185 for enol tautomer. As the Gibbs free energy of the proton solvation in the 1:1 water/methanol medium is unknown, this parameter can be determined through a fitting procedure utilizing Equation (1). The results obtained for keto-form and presented in Table 7 demonstrate that parameters ∆G(H^+^)_sol_ = −267.537(1) kcal mol^−1^, a = [8.9], c = 0.0836(1) are determined with R^2^ = 1.0000 and SE = 0.0013. A comparable accuracy of determination is achieved for the enol form of curcumin, producing ∆G(H^+^)_sol_ = −268.566(2) kcal mol^−1^. Accounting for the dispersion effect at the ωB97XD-D2 theory level influences only the values of the fitted parameters, while the accuracy in reproducing the p*K*_a_ constants for the keto-form remains comparable to that achieved with the LSDA method.

### 2.5. The Global Chemical Activity Descriptors

Analysis of the global chemical reactivity parameters for the keto–enol tautomers of curcumin, as presented in Table 8 and Table 9, reveals that the enol form exhibits higher reactivity compared to the keto form.

This conclusion is supported by a decrease in IP and the energy gap (∆E), alongside an increase in the EA. A lower ∆E value characterizes a softer molecule, which is less stable and thus more chemically reactive. Similarly, a reduced IP suggests an enhanced tendency of the molecule to participate in electron transfer processes. In contrast, a higher EA indicates an increased ability to accept electrons and form the corresponding anionic species. The reactivity of both tautomers is influenced by solvent polarity, with reactivity increasing alongside the dielectric constant (ε). This trend is evidenced by a systematic decrease in the values of IP, ∆E, η, μ, and a concurrent increase in EA, S, and ω as ε increases.

Incorporation of dispersion corrections at the B3LYP-D3 level results in a modest influence on the reactivity descriptors in gas phase, polar (water), and non-polar (benzene) environments. In particular, dispersion interactions exert a relatively weak influence on the chemical hardness η of curcumin, which reflects their resistance to deformation or polarization of the electron cloud under external perturbations such as chemical reagents, dispersion, and solvent interactions. It is noteworthy that the index ω remains virtually unaffected by the inclusion of dispersion for the enol-form, while a slight variation in ω is observed for the keto-form. A similar differentiation in the reactivity of the keto and enol forms is observed with respect to the ∆E, which decreases under the influence of both dispersion interactions and solvent polarity. Notably, in water, ∆E reaches a particularly low value of 3.3478 eV when dispersion is included (compared to 3.3788 eV without dispersion). In contrast, in benzene, an anomalous increase in ∆E is observed upon inclusion of dispersion, from 3.5508 eV to 3.5720 eV. This suggests that dispersion slightly reduces the reactivity of the keto-form in a non-polar benzene environment, in contrast to the aqueous environment, where it enhances reactivity. This effect, however, is negligible for the enol form of curcumin.

The electro-donating (ω^−^) and electro-accepting (ω^+^) powers of the keto–enol forms increase systematically with solvent polarity. Specifically, for the keto (enol) form, ω^−^ increases from 7.1029 (7.5025) eV to 7.7872 (8.2266) eV, and ω^+^ from 2.9686 (3.4473) eV to 3.5225 (4.0244) eV. This trend highlights the significant role of solvent effects in enhancing the antioxidant and antireductant properties of both tautomers. These findings are further corroborated by the electron acceptance (Ra) and donation (Rd) indices, which also increase with ε. For the keto (enol) form, Ra increases from 0.8726 (1.0133) to 1.0354 (1.1830), and Rd from 2.0471 (2.1622) to 2.2529 (2.3709). These values indicate that, in polar media, both tautomers are comparable electron acceptors to fluorine (Ra = 1) and exhibit greater electron-donating ability than sodium (Rd = 1). Consequently, the overall reactivity of both curcumin tautomers in polar solvents is comparable to that of astaxanthin (Ra = 0.94, Rd = 2.10, ω^+^ = 3.21, ω^−^ = 7.27), a compound widely recognized as one of the most effective natural electron acceptors.

Figure S3 presents the frontier orbitals of the keto and enol forms of curcumin in the gas phase, as well as in aqueous and benzene environments, calculated at the B3LYP/6311++G(d,p) level of the theory and the SMD solvation model.

### 2.6. Di-Curcumin Derivative

Thermodynamic and chemical descriptors constitute a valuable tool for predicting the reactivity of both known and synthetic (modeled) chemical compounds. Analysis of the parameter values presented in Table 2 indicates that the reactivity of the hydrogen atom in the methylene group of the keto form, as well as in the hydroxyl group of the enol form of curcumin, is lower—both in the gas phase and in hydrophilic and hydrophobic environments—than that of the hydrogen atoms in the phenolic OH groups. This observation provides a significant indication regarding potential modifications to the original curcumin structure that could enhance its reactivity and eliminate the problem of tautomerization caused by the hydrogen atom of the methylene group—its mobility depends on the dielectric constant and pH of the solvent.

**Figure 5:**
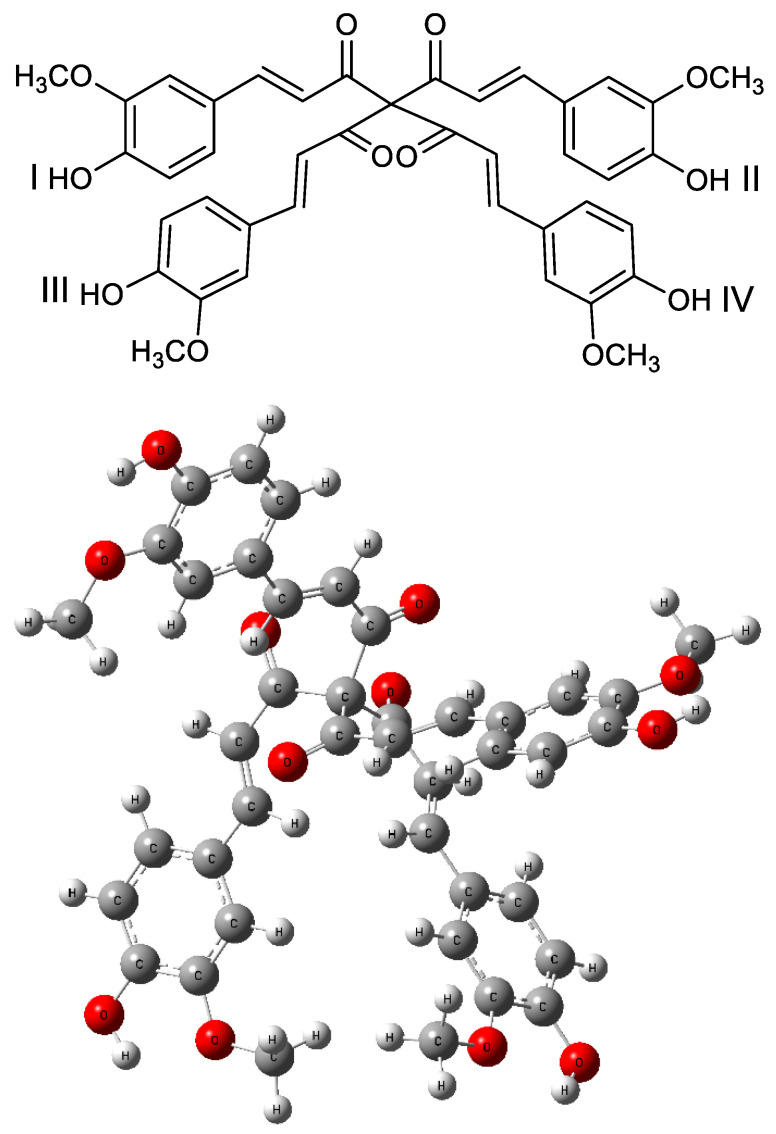
The optimized geometry of the di-curcumin and sites I, II, III, and IV of deprotonation or dehydrogenation. The optimization was performed at the B3LYP/6311++G(d,p) theory level using the SMD solvation model and water medium.

The proposed structural modification involves replacing the two hydrogen atoms of the methylene group in the keto form with two identical moieties present in curcumin, namely, 4-hydroxy-3-methoxyphenyl-prop-1-en-2-one. The modeled compound presented in Figure 5 and referred to as 1,7-bis(4-hydroxy-3-methoxyphenyl)-4,4-bis(3-(4-hydroxy-3-methoxyphenyl)acryloyl)hepta-1,6-diene-3,5-dione—hereafter termed di-curcumin, by analogy with the known class of half-curcuminoids [18]—exhibits enhanced reactivity and anti-radical potential.

Thermodynamic parameters listed in Table 10 demonstrate that the new curcumin analog contains four phenolic OH groups with greater activity than the hydrogen atoms of the methylene group in the keto form and the hydroxyl group in the enol form of the parent compound.

Furthermore, the values of global descriptors also suggest an increased reactivity of the analog compared to the keto form of curcumin (values in parentheses): EA = 2.5791 (2.6052) eV, IP = 5.9128 (5.9841) eV, ∆E = 3.3337 (3.3788) eV, η = 1.6668 (1.6894) eV, S = 0.3000 (0.2960) eV^−1^, χ = −μ = 0.2459 (0.2946) eV, ω = 5.4078 (5.4587) eV, ω^+^ = 3.4932 (3.5225) eV, ω^−^ = 7.7392 (7.8172) eV, Ra = 1.0268 (1.0354), Rd = 2.2304 (2.2529).

Di-curcumin may be synthesized using a method analogous to that employed for the preparation of half-curcuminoids [18], with the primary modification involving the substitution of acetylacetone with 3,3-diacetylopentane-2,4-dione. In the subsequent condensation reaction with 4-hydroxy-3-methoxybenzaldehyde, the formation of di-curcumin is achieved.

## 3. Materials and Methods

The total electronic energies and optimized geometries of the neutral, radical, cationic, anionic, and radical–anionic forms of the keto and enol tautomers of curcumin were calculated in the gas phase and in solvents of varying polarity using DFT method. The calculations employed the Becke three-parameter Lee–Yang–Parr (B3LYP) exchange–correlation functional [50,51], the 6-311++G(d,p) basis set, and the universal Solvation Model based on electron Density (SMD) [48]. The selection of the B3LYP functional and the SMD solvation model was not arbitrary. In test calculations performed for gallic acid [80], the influence of the functional and solvation model on the values of global reactivity descriptors has been investigated. Among the tested combinations, B3LYP/SMD proved to be the most suitable, providing the best reproduction of the optical gap—approximated by the HOMO–LUMO energy difference ∆E.

Initial molecular structures were generated using the GaussView 6.1 graphical interface [81] (Gaussian Inc.,Wallingford, CT, USA), and all quantum chemical computations were performed using the Gaussian 16 software package [81] (Gaussian Inc.,Wallingford, CT, USA). Enthalpies, Gibbs free energies, and zero-point energies of the neutral, anionic, cationic, and radical species were used to calculate thermodynamic descriptors: BDE, PA, ETE, AIP, and PDE [60,61,62] as defined in the Supplementary Materials. In addition, the energies of the highest occupied and lowest unoccupied molecular orbitals (HOMO and LUMO) for the neutral forms were used to evaluate global chemical reactivity descriptors, including: IP, EA, ∆E, μ, χ, η, S ω, ω^−^, ω^+^, Ra, and Rd [63,64,65,66,67,68,69,70] as described also in Supplementary Materials.

Dispersion interactions were considered using three computational DFT approaches: (i) the B3LYP functional with Grimme’s D3 empirical dispersion correction, (ii) the Austin–Frisch–Petersson (AFPD) functional, which incorporates spherical atom dispersion terms [58], and (iii) the ωB97XD functional developed by Head-Gordon and co-workers, which includes Grimme’s D2 dispersion component [59]. These models have been recommended as effective tools for investigating dispersion interactions, as reported in reference [82].

Achieving chemical accuracy in p*K*_a_ calculations presents an inherent challenge, as an error of 1.36 kcal·mol^−1^ in the Gibbs free energy change associated with deprotonation in solution corresponds to a deviation of approximately one p*K*_a_ unit. Consequently, the accurate prediction of p*K*_a_ values requires the use of extended basis sets and advanced computational methodologies [52]. In the present study, we employed the local spin density approximation (LSDA) [49] in conjunction with the quadruple-zeta valence polarized (QZVP) basis set [83], both of which have been previously validated for use with gallic acid in achieving p*K*_a_ values consistent with experimental uncertainties [52]. Furthermore, Equation (1) was utilized to reproduce the experimental p*K*_a_ values of curcumin—p*K*_a_I = 8.54 ± 0.03, p*K*_a_II = 9.30 ± 0.03, and p*K*_a_III = 10.69 ± 0.02 [40]—in a 1:1 water/methanol mixture. Accordingly, the Gibbs free energies of the neutral and anionic forms of the keto–enol tautomers of curcumin were computed in a medium characterized by an effective dielectric constant, ε = (ε_H_2_O + ε_CH_3_OH)/2 = 55.48415. The Gibbs free energies used in these calculations (ΔG_N_), along with the correction term, s = RT·ln(2)·ln(10) = 0.4107 kcal mol^−1^, are summarized in Table 6.

As Equation (1) comprises three adjustable parameters—a, c, and *G*(H^+^)_sol_—where the value of *G*(H^+^)_sol_ remains undetermined for a water–methanol solvent system, simultaneous fitting of all three parameters to the experimental p*K*_a_ values necessitates the implementation of a parameter-scanning methodology. This approach involves the systematic variation in one parameter using a defined step size while calculating fit quality indicators such as the coefficient of determination (R^2^) and/or the standard error (SE) of estimation. The initial parameter estimate is obtained through this procedure and is subsequently refined by reducing the step size—typically by an order of magnitude—and repeating the process iteratively until the optimal parameter values that maximize the fit quality are identified. A scan of the a-parameter for the keto form of curcumin, along with the values of the goodness-of-fit indicators R^2^ and SE, is presented in Table S1.

In the present study, the a-parameter was constrained to the range of values [a] reported in Table 7, which yielded optimal indicators of goodness-of-fit. All calculations were performed using SigmaPlot version 11 software (Systat Software, Inc., San Jose, CA, USA). The resulting fitted parameters were then applied to calculate the p*K*_a_N values for both keto–enol tautomers of curcumin. The accuracy of the model in reproducing the experimental p*K*_a_N(exp) values is quantified by the mean absolute error (MAE) and the normalized mean absolute error (NMAE), defined as follows:

(4) $$\mathrm{MAE}=\frac{1}{3}\sum_{N=I}^{\mathrm{III}}\left|pK_{a}N-pK_{a}N(\exp)\right|\;\;\;\;\;\mathrm{NMAE}=\frac{1}{3}\sum_{N=I}^{\mathrm{III}}\frac{\left|pK_{a}N-pK_{a}N(\exp)\right|}{u_{N}}$$

In this context, u_N_ represents the experimental measurement uncertainties, with u_I_ = 0.03, u_II_ = 0.03, u_III_ = 0.02 [40]. An NMAE value ≤ 1 indicates that the calculated parameters reproduce the experimental data within the bounds of the reported measurement errors.

Given that computations performed at the LSDA/QZVP level of theory are computationally intensive and highly sensitive to the initial molecular geometry, the geometry optimization procedure was divided into two stages. In the first stage, an approximate geometry was obtained at the LSDA/6-311++G(d,p) level of theory in the gas phase. In the second stage, this pre-optimized geometry was used as the starting structure for final optimization at the LSDA/QZVP level within the target solvent environment, employing the SMD solvation model. Each stage involved a complete optimization process, with the lower-level geometry serving as the initial input for the subsequent higher-level calculation.

Although the influence of the basis set on the p*K*_a_ parameter may be significant, its impact on the remaining descriptors is negligible. To assess the impact of employing an extended QZVP basis set on the values of the reactivity parameters, calculations were performed to evaluate its influence on the reproduction of key descriptors—electron affinity (EA), ionization potential (IP), and energy gap (ΔE)—for the keto form of curcumin in the gas phase, as well as in aqueous and benzene environments. The results demonstrate that the use of the extended basis set exerts only a minimal effect and does not alter the interpretation of the compound’s reactivity. Specifically, the following values (in eV) were obtained (values in parentheses correspond to those reported in Table 8): (i) gas phase: IP = 5.9663 (6.0077), EA = 2.3225 (2.3930), ∆E = 3.6471 (3.6148); (ii) aqueous medium: IP = 5.9386 (5.9841), EA = 2.5386 (2.6052), ∆E = 3.4001 (3.3788); (iii) benzene medium: IP = 5.8741 (5.9098), EA = 2.2936 (2.3590), ∆E = 3.5805 (3.5508).

## 4. Conclusions

The findings of this study demonstrate that a comprehensive understanding of curcumin’s reactivity requires consideration of three active sites in both its enol and keto tautomeric forms. The scavenging of free radicals by curcumin may proceed via a multi-step hydrogen atom transfer (HAT) mechanism in hydrophobic environments, and through a multi-step sequential proton loss electron transfer (SPLET) mechanism in which all active centers are involved. These results indicate that the anti-radical activity of curcumin is not solely attributable to its phenolic hydroxyl groups, but also involves the hydrogen atom of the methylene group in the keto form and the hydroxyl group in the enol tautomer. The results obtained indicate that a multistep process enabling the scavenging of three radicals by a single curcumin molecule may be included among the broad spectrum of potential mechanisms by which this compound deactivates free radicals [19,20,21,22,23,24]. An analysis of the PA parameters presented in Table 2, Table 4 and Table 5 reveals that PA_I_ and PA_II_ are consistently lower than PA_III_ across all cases, indicating that deprotonation initially occurs at the phenolic hydrogen atoms, followed subsequently by deprotonation of the methylene or hydroxyl group hydrogen.

The dispersion interaction significantly affects deprotonation at position III of the keto–tautomer, whereas other sites—both in the keto-form, and at positions I, II, and III in the enol form—exhibit only minor sensitivity to dispersion. This suggests that thermodynamic descriptors associated with these sites are only marginally influenced by dispersion effects, which can therefore be reasonably neglected in practical calculations. However, dispersion cannot be ignored when computing the total electronic energy, as its inclusion results in a remarkable energy diminishing, thereby indicating a stabilizing effect on the curcumin molecule. In the limiting case, dispersion may equalize the total energies of the keto and enol forms in the aqueous phase, thereby challenging the prevailing view that the enol form is more stable than the ketone one. The influence of dispersion on the global chemical reactivity descriptors of curcumin is also minimal. In contrast, solvent polarity plays a significant role: the reactivity of curcumin increases with the dielectric constant of the surrounding medium. This observation suggests that the optimal environment for curcumin’s bioactivity is a solvent mixture—such as water/ethanol—where water promotes activation, while ethanol facilitates solubility, a sine qua non condition for initiating its chemical activity and biological functions.

The proposed mixed mechanism of radical deactivation serves as an important guideline for designing new curcumin analogs [17,18,23,26,33,84], which should exhibit at least comparable activity in this respect to the parent compound. For this purpose, strategies that modify the base structure—successfully applied to compounds other than curcumin—can be employed [85,86]. Currently, research is being conducted on novel di-curcuminoid derivatives characterized by enhanced efficiency in free radical scavenging and based on the multi-step deactivation mechanism. The results of these studies will be published shortly.

## Figures and Tables

**Table 1:** The effect of dispersion on the total electronic energy (in Ha) and the energy difference (in kcal mol^−1^) of the keto–enol forms of curcumin. The calculations were performed in the gas phase, water, and benzene medium at the B3LYP, B3LYP-D3, WB97XD-D2, AFPD/6311++G(d,p) theory levels, using the SMD solvation model. The results obtained for the B3LYP functional, which does not take dispersion into account, are given for comparison.

	KETO			ENOL			E_KETO_ − E_ENOL_		
Method	Gas	Water	Benzene	Gas	Water	Benzene	Gas	Water	Benzene
B3LYP	−1263.932619	−1263.962819	−1263.961841	−1263.943446	−1263.943446	−1263.970725	6.79	3.32	5.57
B3LYP-D3	−1263.975214	−1264.005454	−1264.005509	−1263.983896	−1264.008657	−1264.011120	5.45	2.01	3.52
ωB97XD-D2	−1263.495618	−1263.525479	−1263.524857	−1263.501551	−1263.526199	−1263.528925	3.72	0.45	2.55
AFPD	−1262.900324	−1262.930183	−1262.929599	−1262.911555	−1262.936219	−1262.939186	7.05	3.79	6.02

**Table 2:** The thermodynamic descriptors (in kcal mol^−1^) of keto–enol tautomers of curcumin in the gas phase, benzene, and water medium calculated at the B3LYP, B3LYP-D3/6311++G(d,p) theory levels, using the SMD solvation model.

	Keto				Enol			
Parameter	Gas	Benzene	Water	Water D3	Gas	Benzene	Water	Water D3
BDE_I_	81.66	81.83	77.38	77.94	80.59	80.55	75.66	76.16
BDE_II_	81.66	81.93	77.38	77.94	81.03	81.95	76.24	76.10
BDE_III_	88.02	89.92	87.11	88.35	99.88	99.20	100.62	101.18
PA_I_	328.91	96.23	40.87	41.44	327.96	95.60	41.88	42.43
PA_II_	328.91	96.23	40.88	41.44	329.06	96.37	41.96	42.51
PA_III_	332.70	99.23	43.05	44.36	345.24	108.76	45.23	45.74
ETE_I_	67.26	83.22	82.60	82.60	67.13	82.57	79.97	79.83
ETE_II_	67.26	83.33	82.59	82.60	66.47	82.31	80.37	79.69
ETE_III_	69.83	88.31	90.15	90.09	69.15	88.07	101.48	101.54
PDE_I_	232.53	36.91	19.00	19.77	239.90	42.60	22.60	23.17
PDE_II_	232.53	37.01	19.00	19.77	240.34	43.11	23.18	23.10
PDE_III_	238.89	45.00	28.73	30.18	259.19	61.26	47.56	48.18
AIP	163.64	142.55	104.47	104.27	155.20	135.57	99.15	99.09
PA_I_ + ETE_I_	396.17	179.46	123.47	124.04	395.90	178.17	121.75	122.26
PA_II_ + ETE_II_	396.17	179.56	123.47	124.04	395.54	178.68	122.33	122.19
PA_III_ + ETE_III_	402.53	187.55	133.20	134.45	414.39	196.831	146.71	147.28
Mechanism	HAT	HAT	SPLET	SPLET	HAT	HAT	SPLET	SPLET

**Table 3:** Enthalpies (in Ha) of the keto–tautomer of curcumin in neutral (N), radical (R), anionic (A), and cationic (C) forms in water, calculated for the active sites I, II, and III, at the B3LYP, B3LYP-D3/6311++G(d,p) theory levels and SMD solvation model. ∆H (x) = H(x) − H(x,D3) denotes the difference in enthalpy (in kcal mol^−1^) calculated without and with the dispersion effect.

x=	N	R_I_	R_II_	R_III_	A_I_	A_II_	A_III_	C
H(x)	−1263.568101	−1262.945630	−1262.945629	−1262.930124	−1263.116053	−1263.116038	−1263.113531	−1263.362818
H(x,D3)	−1263.610588	−1262.987211	−1262.987212	−1262.970626	−1263.157642	−1263.157642	−1263.152985	−1263.405623
∆H(x)	26.6610	26.0925	26.0937	25.4154	26.0975	26.1069	24.7578	26.8605

**Table 4:** Thermodynamic descriptors (in kcal mol^−1^) of multi-step HAT and SPLET mechanisms of radical deactivation by the keto–tautomer of curcumin and its radical, anionic, and radical–anionic forms. All calculations were carried out in water and benzene using the SMD solvation model at the B3LYP/6311++G(d,p) theory level.

Keto	Water					
Descriptor	NNN	Reaction	ANN	Reaction	AAN	Reaction
BDE_N_	77.38	NNN → RNN	77.24	ANN → ARN	77.72	AAN → AAR
PA_N_	40.87	NNN → ANN	41.81	ANN → AAN	50.55	AAN → AAA
ET_1III_					73.27	AAA → AAR
ETE_II_			81.52	AAN → ARN	59.34	AAR → ARR
ETE_I_	82.60	ANN → RNN	71.56	ARN → RRN	04.50	ARR → RRR
AIP_N_	104.47	NNN → CNN	82.60	ANN → ACN	81.52	AAN → AAC
PDE_N_	19.00	CNN → RNN	40.73	ACN → ARN	42.30	AAC → AAR
**Keto**	**Benzene**					
**Descriptor**	**NNN**	**Reaction**	**RNN**	**Reaction**	**RRN**	**Reaction**
BDE_N_	81.83	NNN → RNN	76.32	RNN → RRN	89.65	RRN → RRR
PA_N_	96.23	NNN → ANN	90.89	RNN → RAN	77.08	RRN → RRA
ETE_N_	83.72	ANN → RNN	83.06	RAN → RRN	110.20	RRA → RRR
AIP_N_	142.55	NNN → CNN	143.24	RNN → RCN	152.90	RRN → RRC
PDE_N_	36.91	CNN → RNN	30.70	RCN → RRN	34.38	RRC → RRR

**Table 5:** Thermodynamic descriptors (in kcal mol^−1^) of multi-step HAT and SPLET mechanisms of radical deactivation by the enol tautomer of curcumin and its radical, anionic, and radical–anionic forms. All calculations were carried out in water and benzene using the SMD solvation model at the B3LYP/6311++G(d,p) theoretical level.

Enol	Water					
Descriptor	NNN	Reaction	ANN	Reaction	AAN	Reaction
BDE_N_	74.89	NNN → RNN	73.59	ANN → ARN	77.81	AAN → AAR
PA_N_	41.88	NNN → ANN	43.23	ANN → AAN	50.35	AAN → AAA
ETE_III_					73.55	AAA → AAR
ETE_II_			76.45	AAN → ARN	85.62	AAR → ARR
ETE_I_	79.11	ANN → RNN	89.75	ARN → RRN	86.98	ARR → RRR
AIP_N_	99.15	NNN → CNN	79.88	ANN → ACN	81.46	AAN → AAC
PDE_N_	21.84	CNN → RNN	39.80	ACN → ARN	42.36	AAC → AAR
**Enol**	**Benzene**					
**Descriptor**	**NNN**	**Reaction**	**RNN**	**Reaction**	**RRN**	**Reaction**
BDE	80.55	NNN → RNN	89.78	RNN → RRN	82.90	RRN → RRR
PA	95.60	NNN → ANN	89.13	RNN → RAN	98.76	RRN → RRA
ETE	82.57	ANN → RNN	98.28	RAN → RRN	81.76	RRA → RRR
AIP	135.56	NNN → CNN	136.39	RNN → RCN	129.13	RRN → RRC
PDE	42.60	CNN → RNN	51.61	RCN → RRN	51.39	RRC → RRR

**Table 6:** The differences ∆G_N_ (in kcal mol^−1^) in Gibbs free energies of neutral curcumin G^0^ and its anionic forms G^−N^ N = I, II, III, including zero-point energy corrections reported in Figure S2. The calculations were performed at the LSDA/QZVP (Keto) and ωB97XD/QZVP (Keto D2) theory levels, using the SMD solvation model and the 1:1 water/methanol medium with ε = (εH_2_O + εCH_3_OH)/2 = 55.48415. The correction to ∆G_N_**,** s = RT∙ln(2)∙ln(10) = 0.4107 kcal mol^−1^ is taken into account, and the influence of dispersion D2 (included in the functional ωB97XD) on ∆G_N_ for the keto form is examined.

	∆G_N_	Keto	Keto D2	s	Enol	s
∆G_I_	G^−I^_sol_ − G^0^_sol_ − s	276.5513	289.4177	0.4107	277.5751	0
∆G_II_	G^−II^_sol_ − G^−I^_sol_ − s	278.0306	290.5193	0	280.0029	0
∆G_III_	G^−III^_sol_ − G^−II^_sol_ − s	287.9236	301.2174	0.4107	307.4928	0

**Table 7:** The parameters a (constrained to value [a]), c, and ∆G(H^+^)_sol_ (kcal mol^−1^) were determined by fitting Equation (1) to the experimental p*K*_a_ values: 8.54 ± 0.03, 9.30 ± 0.03, and 10.69 ± 0.02 for curcumin in a 1:1 water/methanol mixture [40]. These p*K*_a_ values are accurately reproduced by the parameters derived and ∆G_N_ values from Table 6. The quality of the fit is evaluated using statistical indicators: the standard error (SE) of estimation, the coefficient of determination (R^2^), the mean absolute error (MAE in p*K*_a_ units), and the normalized mean absolute error (NMAE).

Parameter	Keto			Keto D2			Enol		
∆G(H^+^)_sol_	−267.68(4)	−267.537(1)	−267.522(2)	−280.5(1)	−280.77(1)	−280.8079(6)	−268.9(1)	−268.546(9)	−268.566(2)
a	[9.0]	[8.9]	[8.89]	[9.0]	[9.2]	[9.23]	[9.0]	[8.8]	[8.81]
c	0.079(1)	0.0836(1)	0.0841(1)	0.078(4)	0.0685(6)	0.0670(1)	0.055(3)	0.0625(2)	0.0621(1)
SE	0.0364	0.0013	0.0020	0.0960	0.0141	0.0008	0.0896	0.0055	0.0013
R^2^	0.9994	1.0000	1.0000	0.9961	0.9999	1.0000	0.9966	1.0000	1.0000
p*K*_a_I	8.5269	8.5393	8.5404	8.5747	8.5463	8.5393	8.5100	8.5414	8.5400
p*K*_a_II	9.3354	9.3009	9.2979	9.2120	9.2876	9.3007	9.3847	9.2949	9.2987
p*K*_a_III	10.6908	10.6891	10.6899	10.7068	10.6935	10.6908	10.6755	10.6918	10.6902
MAE	0.0164	0.0008	0.0008	0.0465	0.0074	0.0007	0.0432	0.0028	0.0005
NMAE	0.5528	0.0328	0.0293	1.6434	0.2660	0.0288	1.5218	0.1023	0.0185

**Table 8:** Chemical activity descriptors (in eV) of the keto-form of curcumin calculated in the gas phase and in solvents of increasing polarity (dielectric constant ε) using the B3LYP and B3LYP-D3 methods with the 6-311++G(d,p) basis set and the SMD solvation model. The values were obtained using Koopmans’ approximation [64], employing the relationships EA ≈ −E_LUMO_, IP ≈ −E_HOMO_.

Medium	ε	EA	IP	∆E	η	S ^2^	χ = −μ	ω	ω^+^	ω^−^	Ra ^3^	Rd ^3^
Gas	1.0000	2.3930	6.0077	3.6148	1.8074	0.2766	4.2003	4.8808	3.0066	7.2069	0.8838	2.0770
Gas D3	-	2.4169	6.0023	3.5854	1.7927	0.2789	4.2096	4.9425	3.0618	7.2714	0.9000	2.0956
Benzene	2.2706	2.3590	5.9098	3.5508	1.7754	0.2816	4.1344	4.8138	2.9686	7.1029	0.8726	2.0471
Benzene D3	-	2.3432	5.9152	3.5720	1.7860	0.2800	4.1292	4.7732	2.9319	7.0611	0.8618	2.0350
Toluene	2.3741	2.3592	5.9076	3.5484	1.7742	0.2818	4.1334	4.8149	2.9700	7.1034	0.8730	2.0472
CLB ^1^	5.6210	2.3720	5.8927	3.5206	1.7603	0.2840	4.1323	4.8503	3.0042	7.1365	0.8831	2.0567
Acetone	20.493	2.3973	5.8986	3.5013	1.7506	0.2856	4.1480	4.9141	3.0589	7.2069	0.8992	2.0770
Ethanol	24.852	2.5440	5.9473	3.4033	1.7017	0.2938	4.2457	5.2964	3.3863	7.2964	0.9954	2.1995
Methanol	32.613	2.5843	5.9634	3.3791	1.6896	0.2959	4.2738	5.4054	3.4797	7.7535	1.0229	2.2346
Water	78.355	2.6052	5.9841	3.3788	1.6894	0.2960	4.2946	5.4587	3.5225	7.8172	1.0354	2.2529
Water D3	-	2.6270	5.9748	3.3478	1.6739	0.2987	5.3009	5.5253	3.5841	7.8850	1.0535	2.2725

^1^ Chlorobenzene. ^2^ In eV^−1^ unit. ^3^ Dimensionless parameters.

**Table 9:** Chemical activity descriptors (in eV) of the enol-form of curcumin calculated in the gas phase and in solvents of increasing polarity (dielectric constant ε) using the B3LYP and B3LYP-D3 methods with the 6-311++G(d,p) basis set and the SMD solvation model. The values were obtained using Koopmans’ approximation [64], employing the relationships EA ≈ −E_LUMO_, IP ≈ −E_HOMO_.

Medium	ε	EA	IP	∆E	η	S ^2^	χ = −μ	ω	ω^+^	ω^−^	Ra ^3^	Rd ^3^
Gas	1.0000	2.4958	5.6722	3.1764	1.5882	0.3148	4.0840	5.2510	3.4075	7.4915	1.0016	2.1591
Gas D3	-	2.4956	5.6700	3.1745	1.5872	0.3150	4.0828	5.2510	3.4080	7.4908	1.0018	2.1589
Benzene	2.2706	2.4980	5.6123	3.1143	1.5572	0.3211	4.0552	5.2802	3.4473	7.5025	1.0133	2.1622
Benzene D3	-	2.4988	5.6107	3.1119	1.5559	0.3213	4.0548	5.2833	3.4504	7.5052	1.0142	2.1630
Toluene	2.3741	2.5002	5.6113	3.1111	1.5555	0.3214	4.0557	5.2872	3.4538	7.5094	1.0152	2.1642
CLB ^1^	5.6210	2.5407	5.6121	3.0713	1.5357	0.3256	4.0764	5.4103	3.5641	7.6405	1.0477	2.2020
Acetone	20.493	2.5832	5.6243	3.0411	1.5206	0.3288	4.1037	5.5376	3.6758	7.7796	1.0805	2.2421
Ethanol	24.852	2.6664	5.6562	2.9897	1.4949	0.3345	4.1613	5.7920	3.8982	8.0595	1.1459	2.3227
Methanol	32.613	2.6898	5.6689	2.9791	1.4896	0.3357	4.1794	5.8633	3.9598	8.1392	1.1640	2.3457
Water	78.355	2.7157	5.6885	2.9728	1.4864	0.3364	4.2021	5.9397	4.0244	8.2266	1.1830	2.3709
Water D3	-	2.7154	5.6869	2.9715	1.4857	0.3365	4.2012	5.9397	4.0249	8.2260	1.1831	2.3707

^1^ Chlorobenzene. ^2^ In eV^−1^ unit. ^3^ Dimensionless parameters.

**Table 10:** The thermodynamic descriptors (in kcal mol^−1^) of di-curcumin in the water medium calculated at the B3LYP/6311++G(d,p) theory levels, using the SMD solvation model.

N	BDE_N_	PA_N_	ETE_N_	PDE_N_	PA_N_ + ETE_N_	AIP
I	77.59	41.04	82.64	19.90	123.69	103.79
II	77.79	40.79	83.09	20.10	123.88	
III	77.46	41.34	82.21	19.77	123.55	
IV	77.73	40.66	83.17	20.05	123.83	

## Data Availability

The original contributions presented in this study are included in the article/Supplementary Materials. Further inquiries can be directed to the corresponding author.

## Supplementary Materials

The following supporting information can be downloaded at: https://www.mdpi.com/article/10.3390/ijms262110374/s1.

## Abbreviations

The following abbreviations are used in this manuscript:

AIP	Adiabatic Ionization Potential
BDE	Bond Dissociation Enthalpy
CLB	Chlorobenzene
CPET	Concerted Proton-Electron Transfer
DFT	Density Functional Theory
DMSO	Dimethyl Sulfoxide
EA	Electron Affinity
ETE	Electron Transfer Enthalpy
HAT	Hydrogen Atom Transfer
HOMO	Highest Occupied Molecular Orbital
IP	Ionization Potential
LUMO	Lowest Occupied Molecular Orbital
MAE	Mean Absolute Error
NMAE	Normalized Mean Absolute Error
PA	Proton Affinity
PCET	Proton-Coupled Electron Transfer
PDE	Proton Dissociation Enthalpy
RAF	Radical Adduct Formation
SE	Standard Error
SET	Single Electron Transfer
SET-PT	Single Electron Transfer Followed by Proton Transfer
SPLET	Sequential Proton Loss Electron Transfer
TD-DFT	Time Dependent Density Functional Theory
VIP	Vertical Ionization Potential
