# Supplementary material for: Reactivity of Curcumin: Theoretical Insight from a Systematic Density Functional Theory-Based Review
Source: Int J Mol Sci. 2025 Oct 24;26(21):10374. doi: 10.3390/ijms262110374 (PMC12610026; doi:10.3390/ijms262110374)
Supplement: Supplementary file 1 [file ijms-26-10374-s001.zip › ijms-3876350-supplementary.pdf]

Supplementary Materials

Reactivity of Curcumin: Theoretical Insight from a Systematic Density Functional Theory-Based Review

Marcin Molski

Quantum Chemistry Department
Faculty of Chemistry
Adam Mickiewicz University
ul. Uniwersytetu Poznańskiego 8, 61-614 Poznań, Poland
mamolski@amu.edu.pl

**Keto**		**Enol**	
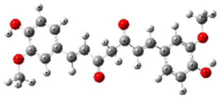	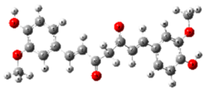	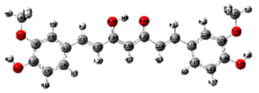	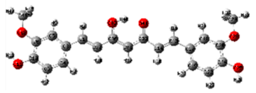
$E_1 = -1263.932619$<br>$\Delta E_{11} = 0$	$E_2 = -1263.924919$<br>$\Delta E_{21} = 4.83$	$E_1 = -1263.943446$<br>$\Delta E_{11} = 0$	$E_2 = -1263.935580$<br>$\Delta E_{21} = 4.94$
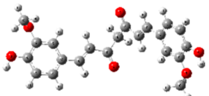	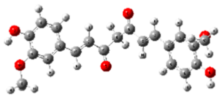	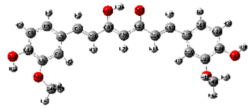	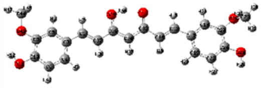
$E_3 = -1263.924103$<br>$\Delta E_{31} = 5.34$	$E_4 = -1263.922812$<br>$\Delta E_{41} = 6.15$	$E_3 = -1263.934299$<br>$\Delta E_{31} = 5.74$	$E_4 = -1263.933508$<br>$\Delta E_{41} = 6.24$
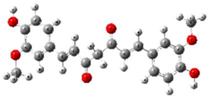	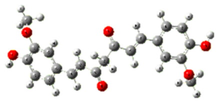	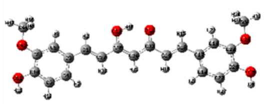	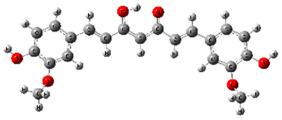
$E_5 = -1263.917212$<br>$\Delta E_{51} = 9.67$	$E_6 = -1263.913643$<br>$\Delta E_{61} = 11.91$	$E_5 = -1263.927754$<br>$\Delta E_{51} = 9.85$	$E_6 = -1263.924817$<br>$\Delta E_{61} = 11.69$
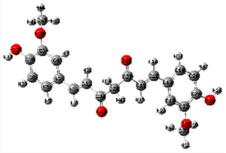	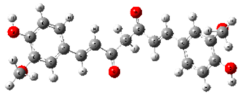	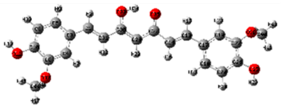	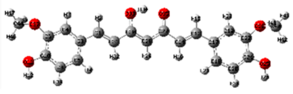
$E_7 = -1263.913423$<br>$\Delta E_{71} = 12.05$	$E_8 = -1263.913169$<br>$\Delta E_{81} = 12.21$	$E_7 = -1263.924245$<br>$\Delta E_{71} = 12.05$	$E_8 = -1263.923630$<br>$\Delta E_{81} = 12.43$
	**Dienol**		
	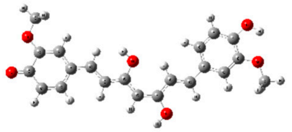		
$\Delta E_{01} = 24.15$	$E_0 = -1263.894129$		$\Delta E_{01} = 30.95$

Figure S1. The optimized geometries of the conformers and rotamers corresponding to the lowest total electronic energies (in Ha) of the keto–enol tautomers of curcumin obtained through gas phase calculations at the B3LYP/6-311++G(d,p) level of theory. The relative energy values, ΔE_{1i}, of the i-th tautomer (in kcal mol^{-1}) are also presented.

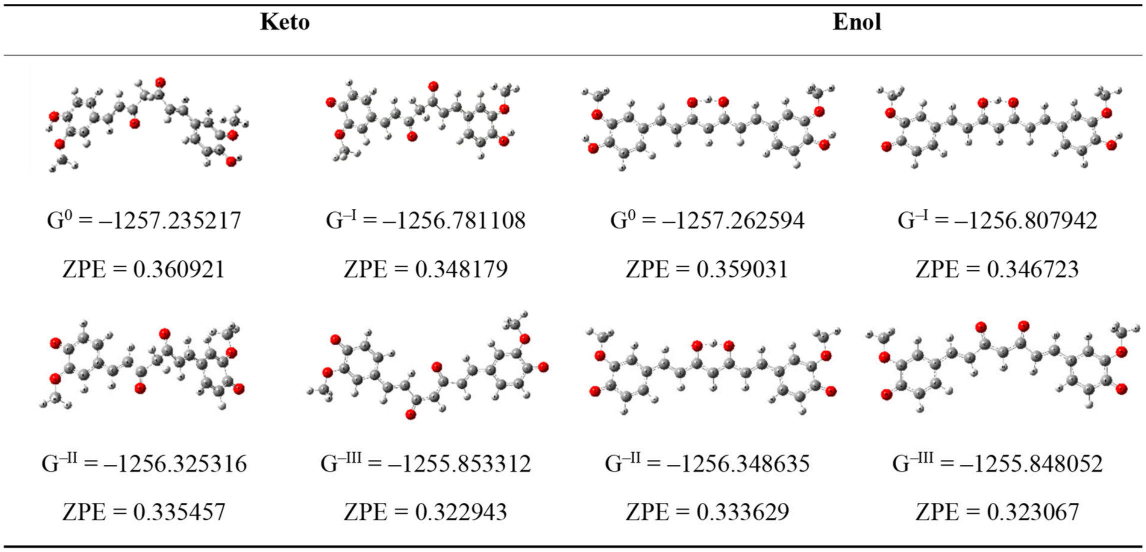

Figure S2. The Gibbs free energies and zero-point energies (ZPE) (in Ha) of neutral curcumin G^0 and its anionic forms G^{-N} N = I, II, III. The calculations were performed at the LSDA/QZVP theory levels, using the SMD solvation model and the 1:1 water/methanol medium with $\varepsilon = (\varepsilon H_2O + \varepsilon CH_3OH)/2 = 55.48415$.

**HOMO**	$\xrightarrow{\Delta E}$	**LUMO**
	**Keto**	
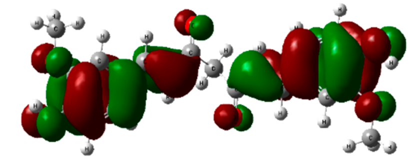		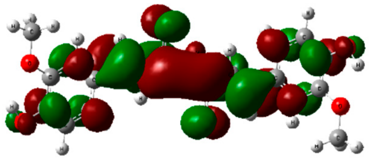
GAS IP = 6.0077	3.6148	GAS EA = 2.3930
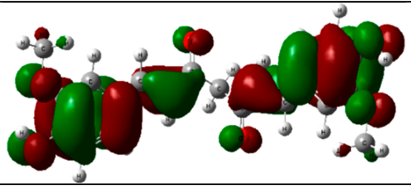		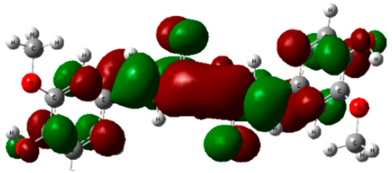
H2O IP = 5.9841	3.3788	H2O EA = 2.6052
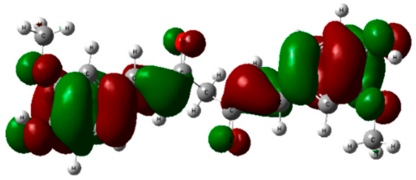		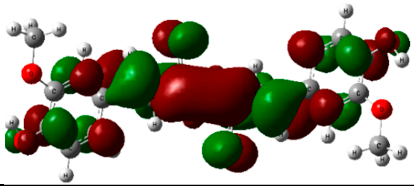
C6H6 IP = 5.9098	3.5508	C6H6 EA = 2.3590
	**Enol**	
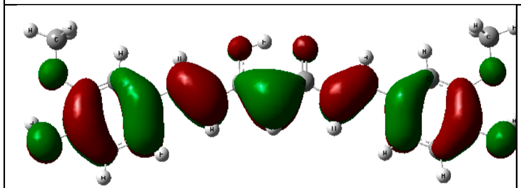		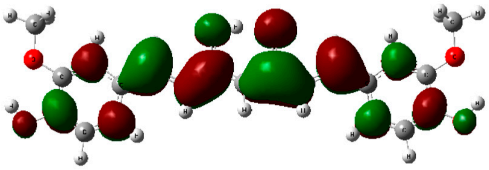
GAS IP = 5.6722	3.1764	GAS EA = 2.4958

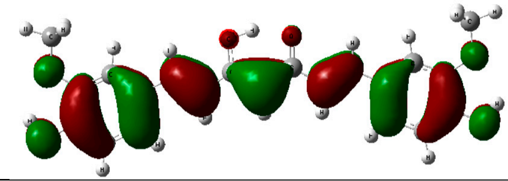		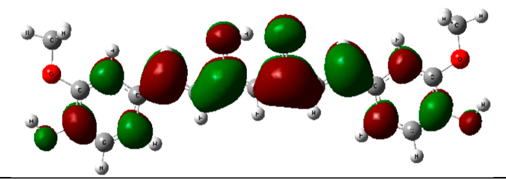
H2O IP = 5.6885	2.9728	H2O EA = 2.7157
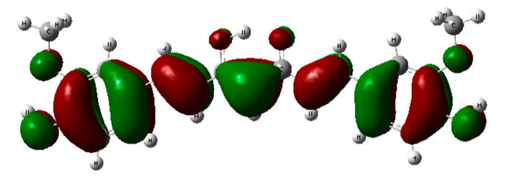		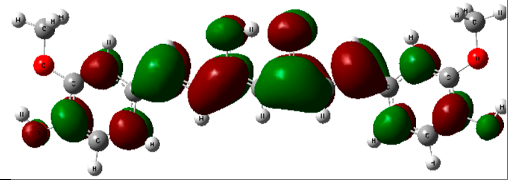
C6H6 IP = 5.6123	3.1143	C6H6 EA = 2.4980

Figure S3. The electron affinity EA = – E_{LUMO}, ionization potential IP = – E_{HOMO}, energy gap ΔE = IP – EA (in eV), and frontier orbitals of the keto and enol forms of curcumin in the gas phase, as well as in aqueous and benzene environments, calculated at the B3LYP/6311++G(d,p) level of the theory and the SMD solvation model. The calculations for the molecular orbitals were accomplished with isovalue 0.02.

Table S1. A scan of the a-parameter for the keto form of curcumin, along with the values of the goodness-of-fit indicators R^2 and SE, obtained by fitting the parameters of Equation (1) to the experimental pK_a values of curcumin. During the fitting process, the parameter a was constrained to the value [a].

[a]	R^2	SE	[a]	R^2	SE
5	0.9602	0.3076	9.1	0.9975	0.0766
6	0.9658	0.2853	9.0	0.9994	0.0364
7	0.9740	0.2484	8.9	1.0000	0.0013
8	0.9873	0.1740	8.8	0.9996	0.0293
9	0.9994	0.0364	8.7	0.9987	0.0560
10	0.7780	0.7265	8.6	0.9973	0.0795

1. Thermodynamic Descriptors and Scavenging Mechanisms

Free radicals can be deactivated by the R─H compound via the three fundamental mechanisms specified below.

HAT (*Hydrogen Atom Transfer*)

$$R\text{—}H + X^{\bullet} \rightarrow R^{\bullet} + X\text{—}H$$

This mechanism is widely applied in processes in which the hydrogen atom is transferred from the basic compound to the reactive intermediate. The antiradical potency of a compound is related to the low bond dissociation enthalpy (BDE) of the N–H bond in amines, S–H in organosulfur compounds, O–H in polyphenols, alcohols, carboxylic acids and O=C–H in aldehydes. Hence, crucial for calculating the BDE parameter is reaction

$$R\text{—}H \rightarrow R^{\bullet} + H^{\bullet}$$

initiating the HAT process, which allows defining the BDE parameter [60]

$$BDE = H(R^{\bullet}) + H(H^{\bullet}) - H(R\text{-}H)$$

in which $H(R^{\bullet})$, $H(H^{\bullet})$ and H (R─H) denote enthalpies of the radical, hydrogen atom, and the neutral molecule. Utilizing the notation introduced in the main manuscript, the above formula can be generalized to a form applicable to the description of multistep dehydrogenation processes

$$BDE_{I} = H(RNN) + H(H^{\cdot}) - H(NNN)$$

$$BDE_{II} = H(RRN) + H(H^{\cdot}) - H(RNN)$$

$$BDE_{III} = H(RRR) + H(H^{\cdot}) - H(RRN)$$

SPLET (*Sequential Proton Loss Electron Transfer*)

$$R─H \rightarrow R^{-} + H^{+}$$

$$R^{-} + X^{\bullet} + H^{+} \rightarrow R^{\bullet} + X─H$$

The second step of the reaction above specified is initiated by the reaction

$$R^{-} \rightarrow R^{\bullet} + e^{-}$$

The SPLET process is therefore characterized by the proton affinity (PA) and electron transfer enthalpy (ETE) calculated according to the formulae [60]

$$PA = H(R^{-}) + H(H^{+}) - H(R\text{-}H)$$

$$ETE = H(R^{\cdot}) + H(e^{-}) - H(R^{-})$$

If the reactions specified above are not sequential and involve the concerted shift of a single electron and a single proton they are called Concerted Proton-Electron Transfer (CPET) [61]. This pathway belongs to the wide class of a Proton-Coupled Electron Transfer (PCET) [62], which involves the shift of electrons and protons from one molecule (atom) to another. The aforementioned formulas can be adapted to characterize a multistep deprotonation process coupled with electron transfer

$$PA_{I} = H(ANN) + H(H^{+}) - H(NNN)$$

$$PA_{II} = H(AAN) + H(H^{+}) - H(ANN)$$

$$PA_{III} = H(AAA) + H(H^{+}) - H(AAN)$$

$$ETE_{III} = H(AAR) + H(e^{-}) - H(AAA)$$

$$ETE_{II} = H(ARR) + H(e^{-}) - H(AAR)$$

$$ETE_{I} = H(RRR) + H(e^{-}) - H(ARR)$$

SET-PT (*Single Electron Transfer Followed by Proton Transfer*)

$$R─H + X^{\bullet} \rightarrow R─H^{\bullet+} + X^{-}$$

$$R─H^{\bullet+} \rightarrow R^{\bullet} + H^{+}$$

This scenario involves the transfer of an electron from the parent compound

$$R─H \rightarrow R─H^{\bullet+} + e^{-}$$

generating a cation radical $R─H^{\bullet+}$ and, in the next step, the proton transfer from the cation radical producing parent agent in the radical form $R^{\bullet}$. These two stages are characterized by the adiabatic ionization potential (AIP) and the proton dissociation enthalpy (PDE) calculated from the formulae [60]

$$AIP = H(R\text{-}H^{\bullet +}) + H(e^-) - H(R\text{-}H)$$

$$PDE = H(R^{\bullet}) + H(H^+) - H(R\text{-}H^{\bullet +})$$

Here, $H(R{-}H^{\bullet +})$, $H(e^-)$, $H(H^+)$, and $H(R{-}H)$ represent the enthalpies of the cation, electron, proton, and the parent compound, respectively. AIP is determined using the enthalpies of the optimized cationic state and the optimized neutral molecule. The Vertical Ionization Potential (VIP) defined as the difference between the energy of the optimized neutral structure and that of the corresponding cationic system at the neutral geometry is not considered in this work. For multi-step processes the SET-PT mechanism is characterized by an exemplary set of equations in which N can be replaced by A or K, as shown in Tables 4 and 5

$$AIP_I = H(CNN) + H(e^-) - H(NNN)$$

$$AIP_{II} = H(NCN) + H(e^-) - H(NNN) = AIP_I$$

$$AIP_{III} = H(NNC) + H(e^-) - H(NNN) = AIP_{II}$$

$$PDE_I = H(RNN) + H(H^+) - H(CNN)$$

$$PDE_{II} = H(NRN) + H(H^+) - H(NCN)$$

$$PDE_{III} = H(NNR) + H(H^+) - H(NNC)$$

RAF (*Radical Adduct Formation*)

In this scenario, radicals are deactivated in the reaction

$$R{-}H + X^{\bullet} \rightarrow R{-}XH^{\bullet}$$

which is characterized by the enthalpy or Gibbs free energies formation calculated for a given radical and an antioxidant considered. Hence, this pathway is characterized by the descriptor depending on the radical type. Consequently, it will not be considered here.

In calculations of the BDE, PDE, AIP, ETE, and PA, we used the values of the electron, proton and hydrogen enthalpies in the gas phase and different solvents, recommended by Rimarčik et al. [60].

2. Global Chemical Reactivity Descriptors

An important source of information on the reactivity of chemical compounds is the difference in the energies of the HOMO and LUMO frontier orbitals. The shape of these orbitals (presented in Figure S3), as well the energy gap (usually expressed in electron volts eV]

$$\Delta E = E_{LUMO} - E_{HOMO}$$

have an impact on the chemical reactivity of compounds. A large energy difference defines a *hard* molecule that is more stable and less active, while a small energy gap defines a *soft* molecule that is less stable and more reactive. Based on the energy of the LUMO and HOMO orbitals, as well as the Koopmans' theorem [63] for the closed shell molecules, one may define the global activity descriptors [64,65,66,67,68,69], which model the physicochemical properties of chemical compounds [70]. The most important chemical activity descriptors are specified below.

Ionization potential

$$IP = -E_{HOMO}$$

It describes the minimum energy required to remove an electron from the molecule's HOMO orbital and move it to infinity. The small IP value indicates a greater tendency of the molecule to participate in the chemical reaction related to electron transfer. The best radical scavengers are endowed with low IP values.

Electron affinity

$$EA = -E_{LUMO}$$

It expresses the ability of a molecule to accept an electron and produce an anion. Due to the fact that radicals scavenging might act either via donating or accepting electrons, the EA descriptor is useful to characterize the capacity of a compound to accept electrons.

Chemical hardness

$$\eta \approx \frac{E_{LUMO} - E_{HOMO}}{2}$$

It describes a molecule with a low susceptibility to deformation or polarization of the electron cloud under the influence of external factors, e.g., reagents.

Chemical softness

$$S \approx \frac{1}{E_{LUMO} - E_{HOMO}}$$

The inverse of chemical hardness – the S-descriptor characterizes molecules with the high susceptibility to deformation and polarization of the electron cloud.

Chemical potential

$$\mu \approx \frac{E_{LUMO} + E_{HOMO}}{2}$$

Employed to describe the thermodynamic activity of substances and used, for example, in the derivation of the phase equilibrium constants.

Electronegativity

$$\chi = -\mu \approx -\frac{(E_{LUMO} + E_{HOMO})}{2} = \frac{EA + IP}{2}$$

It characterizes a tendency to attract electrons that create the chemical bond. The common electron pair is shifted towards the atom having a high value of χ, which is accompanied by the formation of a polar or ionic bond.

Electrophilicity index

$$\omega \approx \frac{\chi^2}{2\eta} = \frac{(E_{LUMO} + E_{HOMO})^2}{4(E_{LUMO} - E_{HOMO})}$$

This descriptor was introduced by Robert Parr and coworkers [68]. It measures the energy change of an electrophilic reagent (acceptor) when it is saturated with electrons provided by another reagent (donor). Higher values of ω characterize a strong electrophile, whereas a strong nucleophile is described by lower values of ω.

Electro-donating and electro-accepting power

A radical scavenger (RS) can act in two ways: either by donating electrons to, or accepting electrons from a radical (R)

$$RS + R \rightarrow RS^{+} + R^{-} \qquad RS + R \rightarrow RS^{-} + R^{+}$$

Following this fact, Gázquez et al. [69] introduced new descriptors characterizing the electro-donating ω^- and electro-accepting ω^+ power of RS, defined in the following manner

$$\omega^- = \frac{(3IP + EA)^2}{16(IP - EA)} \qquad \omega^+ = \frac{(IP + 3EA)^2}{16(IP - EA)}$$

To characterize the relative electro-accepting (donating) power of an arbitrary radical scavenger X, Martinez [70] introduced the acceptance Ra and donation Rd indexes

$$Ra = \frac{\omega_X^+}{\omega_F^+} \qquad Rd = \frac{\omega_X^-}{\omega_{Na}^-}$$

defined by the IP and EA for the F and Na atoms. They represent a good electron acceptor (F) and a good donor (Na), respectively. In the calculations, the well-known experimental values [24] for F: IP = 17.42282, EA = 3.4011898 eV, and for Na: IP = 5.1391, EA = 0.547926 eV are employed. The descriptors Ra and Rd are useful for classifying any compound X in terms of its electron donating (accepting) capacity. Hence, Ra and Rd can be applied to characterize the antiradical capacity of X in terms of its electro-donating (accepting) power with respect to the F and Na atoms, taken as the reference points [70].
